# The Neuroprotective Effects of Moderate and Regular Caffeine Consumption in Alzheimer's Disease

**DOI:** 10.1155/2021/5568011

**Published:** 2021-08-17

**Authors:** Xiangyu Zhou, Lin Zhang

**Affiliations:** ^1^School of Food Science and Nutrition, University of Leeds, Leeds, West Yorkshire LS2 9JT, UK; ^2^The Key Laboratory for Special Medical Food Process in Hunan Province, Central South University of Forestry and Technology, Changsha, Hunan 410004, China

## Abstract

The increasing numbers of elderly Alzheimer's disease (AD) patients because of a steady increase in the average lifespan and aging society attract great scientific concerns, while there were fewer effective treatments on AD progression due to unclear exact causes and pathogenesis of AD. Moderate (200-500 mg/d) and regular caffeine consumption from coffee and tea are considered to alleviate the risk of AD and have therapeutic potential. This paper reviewed epidemiological studies about the relationship of caffeine intake from coffee or/and tea with the risk of AD and summarized the caffeine-related AD therapies based on experimental models. And further well-designed and well-conducted studies are suggested to investigate the optimal dosages, frequencies, and durations of caffeine consumption to slow down AD progression and treat AD.

## 1. Introduction

AD is the most common cause of dementia, which is associated with the physical deterioration of the brain tissue, leading to greater cognitive malfunctions than those of normal brain aging [1]. The incident rate of AD increases dramatically with age, only 2% among 65 years of age, 12.7% among 90 years of age, and 21.2% among 95 years of age, respectively [2]. And cognitive impairment, characterized by rapid memory and attention decline, is the high-risk factor for AD [1]. Because cognitive impairment is likely to progress to AD at a rate of 10% quicker than normal cognitive people at the same age [3], cognitive decline is regarded as a preclinical marker for early dementia. Thus, lowering cognitive decline also indicates a reduction of the risk of AD [4]. Furthermore, cognitive disorders include dementia, cognitive impairment, and cognitive decline [5].

AD is the consequence of the complex interplay between the genetic and environmental factors, including medical history of diseases and dietary habits [6]. Currently, there are limited efficient pharmacological therapies in reversing the cognitive deterioration and slowing down the progression of AD [7]. And the permitted six drugs only provide temporary and incomplete symptomatic relief accompanied by severe side effects [8]. Therefore, clinicians considered modifiable risk factors for brain function preservation, such as lifestyle, obesity, diabetes, and hypertension [5]. Because those nonpharmacological interventions are easy to achieve, acceptable, cheap, and without negative consequences at routine levels, they could help reduce healthcare costs at population levels as good prophylaxis of AD [9]. Concerning aspects relating to lifestyle, multiple studies have examined the potential role of phytochemicals in preventing and slowing down progressive pathogenic changes in AD, including flavonoids, phenolic acids, carotenoids, curcumin, resveratrol, and some alkaloids (in the comprehensive reviews [10, 11]). Among them, the effects of caffeine seem to be well researched and documented [12].

Caffeine (1,3,7-trimethylxanthine), a purine alkaloid, is one of the most common and widely consumed psychoactive stimulants daily, exerting its functions on CNS to help antifatigue, increase concentration, and trigger the arousal of neurons after short-term consumption [13]. Because chronic low doses of caffeine have been reported to protect against CNS hypoxia and ischemia in rats [14] and gerbils [15, 16], whereas acute caffeine administration exacerbated ischemic neuronal damage in rats with forebrain ischemia produced by bilateral carotid occlusion plus hypotension [14], caffeine may have neuroprotective effects; thus, it is reasonably hypothesized that regular caffeine consumption at a low dose for the long term could help prevent AD.

This article is initially aimed at examining the potential role of constant caffeine consumption in AD development based on human studies and treating AD based on experimental studies. The second aim is to recommend caffeine dosages, frequencies, and durations that may be beneficial.

## 2. Caffeine in AD: Human Study

Coffee and tea are the two most popular drinks worldwide and are the leading global dietary sources of caffeine [13]. Although caffeine contents vary in a cup of coffee in different studies due to various serving sizes (50-190 ml), types of coffee beans (Arabica or Robusta), preparation methods (boiled or filtered), and serving types (decaffeinated or Italian), the mean caffeine content is generally 90 mg per 230 ml of coffee (a regular cup of coffee is 230 ml) [17]. Caffeine amounts also vary in a cup of different types of tea. Fresh tea leaves should undergo the diverse degree of fermentation and oxidation of polyphenols during manufacturing; therefore, 100 ml of nonfermented green tea has 15 mg of caffeine on average, and semifermented oolong tea and fermented black tea have 17 mg of caffeine per 100 ml on average (a regular cup of tea is 100 ml) [18]. If coffee or/and tea consumption could provide appropriate dosages of caffeine to modify the progression of a neurodegenerative disorder that may evolve many years before the emergence of visible clinical symptoms, as appears to be the case with AD, without side effects, they may be recommended as a daily natural complementary therapy for lowering the risk of AD and slowing down the progression of AD.

This article summarizes 15 human studies including case-control studies, cohort studies, cross-sectional studies, and meta-analyses in Table 1, to access the possible effects of caffeine from coffee or/and tea on AD and suggest optimal dosages, frequencies, and durations of coffee and tea consumption.

### 2.1. Method

(1) If the selected human studies defined neither the exact caffeine doses in a cup of coffee and tea nor the exact volume of a cup, the caffeine amounts were determined by 90 mg caffeine per cup of coffee (a regular cup of coffee is 230 ml) [17], 15 mg caffeine per cup of green tea, and 17 mg caffeine per cup of black/oolong tea (a regular cup of tea is 100 ml) [18]. (2) The average amounts of daily coffee and tea consumption by Canadians were determined by Conway [19] and Lindsay et al. [20], respectively. (3) Because Westerners consume more black tea and rarer green tea than Easterners [21], if the particular types of tea were not identified in the study from the West, tea consumption refers to the mean caffeine amounts of black tea. (4) Tea consumption based on the Eastern study refers to the majority of consumed tea types.

### 2.2. Caffeine

A retrospective and matched-pair case-control study reported that AD patients only consumed an average of 73.9 ± 97.9 mg/d caffeine as compared to healthy control cases who had 198.7 ± 135.7 mg/d during the last 20 years preceding AD diagnosis. And caffeine exposure during this period could lower the risk of AD significantly with an OR of 0.40 (95% CI = 0.25-0.67). Daily caffeine intake was sourced from instant coffee (60 mg caffeine/142 ml), decaffeinated coffee (3 mg caffeine/142 ml), tea leaf (30 mg caffeine/142 ml), instant tea (20 mg caffeine/142 ml), and cola drinks (18 mg caffeine/170 ml) [22]. However, another nested case-control study observed that midlife caffeine intake from coffee (137 mg/227 ml), tea (47 mg/227 ml), and cola (46 mg/340 ml) was not significantly associated with the risk of late-life AD (25 years later). But the highest levels of caffeine consumption (411.0–1872.5 mg/d) were related to a lower OR of having any of the neuropathological lesion types at autopsy as compared to lower caffeine intake (≤137.0 mg/d) (multivariable-adjusted OR = 0.45, 95% CI = 0.23-0.89, and *P* = 0.04). And the adjusted mean caffeine intake among decedents with AD lesions was 279 mg as compared to 333 mg among those without lesions (*P* = 0.10) [23]. The 2010 meta-analyses of 11 studies reported that caffeine intake could reduce the risk of AD, with the summary RR of 0.83 (95% CI = 0.32–2.15, *I*^2^ = 40.5%) [24].

### 2.3. Coffee

After a 21-year-long period of follow-up observations, the Finnish cohort study reported that participants who consumed 690 to 1150 mg/d of coffee (270 to 450 mg/d caffeine) at midlife had a decreased risk of late-life AD by 58% significantly compared with those drinking 0 to 460 mg/d of coffee (0 to 180 mg/d caffeine, reference). Tea consumption was not associated with a decreased risk of AD later in life, partially because the majority of participants (60.5%) in this study did not drink tea, making statistical power low [25]. A large-scale population-based prospective cohort study among Canadians aged above 65 years consistently reported that daily coffee consumption (243 mg/d caffeine [26]) reduced the risk of AD by 31% during a 5-year follow-up, while daily tea drinking (64 mg/d caffeine [19]) was not associated with lowering AD risk (OR = 1.12, 95% CI = 0.78-1.61) [20]. Furthermore, a multiethnic cohort study among persons aged above 45 years old reported that, during an average of 16.2 years of follow-up, higher coffee intake (above 2 cups/d, above 180 mg/d caffeine) lowered the risk for all-cause death, with an HR of 0.82, as compared to 1 cup/d (HR = 0.88, 95% CI = 0.85–0.91). But only lower coffee consumption (1 cup/d, 90 mg/d caffeine) had a marginally positive association with the risk of AD (HR = 0.90, 95% CI = 0.71–1.14) as compared to a negative effect by higher coffee consumption (above 2 cups/d, 180 mg/d caffeine) [27]. Another large population-based cohort study of old Swedish adults (mean age of 83.2 years) reported that there were no associations of coffee consumption (177 mg/d caffeine) and risk of dementia during a mean follow-up of 12.6 years [28]. However, the 2007 meta-analyses of 4 observational studies reported that coffee consumption could significantly reduce AD risk in comparison with nonconsumers with a pooled risk estimate of 0.73 (95% CI = 0.58–0.92), in a highly significant heterogeneity (chi-squared: 13.6, *P* < 0.01) [29].

### 2.4. Tea

A national population-based prospective nested case-control study on illiterate elderly Chinese subjects reported a significant inverse relationship between dietary habits of tea drinking and cognitive decline (OR = 0.82, 95% CI = 0.68-1.00, and *P* = 0.0468) [30]. And the community-based cross-sectional study among elderly Japanese subjects aged above 70 years also reported an inverse dose-dependent response between green tea consumption and prevalence of cognitive impairment. Subjects who consumed over 200 ml/d green tea (30 mg/d caffeine) and 100 ml/d (15 mg/d caffeine) had a significantly lower cognitive impairment risk by 54% and by 38%, respectively, as compared to below 300 ml/wk (45 mg/wk caffeine, reference) (*P* = 0.0006). However, a weaker association was observed for black or oolong tea consumption with the risk of cognitive impairment, and there was a null association between coffee consumption and the risk of cognitive impairment [31]. On the contrary, in the Singaporean cross-sectional study, participants habitually consumed vastly more black or oolong tea than green tea. Thus, the more inverse relation of black or oolong tea consumption with cognitive impairment was found as compared to green tea. And a higher intake corresponded to a lower risk of cognitive impairment, with an OR of 0.46 (95% CI = 0.31-0.68) for above 215 ml/d (37 mg/d caffeine) and an OR of 0.55 (95% CI = 0.38-0.79) for occasional intake. No significant associations were found between coffee intake and cognitive status [4]. And another Singaporean cross-sectional study among Chinese elders aged above 55 years reported that total tea consumption (34 mg/d caffeine) was related to better performance on global cognition (MMSE) (*B* = 0.055, SE = 0.026, and *P* = 0.03) and memory improvement (*B* = 0.031, SE = 0.012, and *P* = 0.01). The neuroprotective effects of tea consumption on cognitive function were not limited to a particular tea type. As 45.8% of participants consumed Chinese black/oolong tea, 37.6% consumed English black tea and 21.6% had green tea. However, no association was found between coffee intake and cognitive function, as well as a decrease in AD risk [32]. Another cross-sectional study among elderly Norwegians (aged 70-74 years) observed that habitual tea consumers who consumed a mean value of 222 ml/d (37 mg/d caffeine) black tea during the previous years had better cognitive performance than nonconsumers, examined by cognitive tests other than MMSE. And the sharpest dose-response effect of tea was up to 200 ml/d on cognitive performance, after which it tended to plateau [21]. Meanwhile, a meta-analysis of 26 studies (predominately Chinese studies) showed that tea drinking was significantly associated with a decreased risk of cognitive disorders in the elders (OR = 0.65, 95% CI = 0.58-0.73, and *I*^2^ = 78.8%) as compared to nonconsumers or rare consumers, partially owning to neuroprotective effects of caffeine components in tea. There were elusive findings of the relationship between tea intake and AD in the subgroup analysis (OR = 0.88, 95% CI = 0.65-1.12) due to the lack of included studies, especially non-Chinese studies [33]. On the contrary, a meta-analysis of 20 studies concluded 5 AD-related studies, which reported that caffeine intake from coffee or tea was not significantly associated with the risk of AD in the random-effects model among elderly participants, with a subtotal OR/RR of 0.78 (95% CI = 0.50-1.22, *I*^2^ = 71.0%) [5].

### 2.5. Discussions on Human Studies

The current studies suggested that caffeine intake may be associated with a lower risk of cognitive disorders including AD, cognitive impairment, and cognitive decline, despite the presence of some inconsistent results. And the neuroprotective effects of caffeine were closely tied to the appropriate frequencies and dosages of consumption. According to Cappelletti et al., low caffeine intake is less than 200 mg/d, moderate caffeine intake is between 200 and 500 mg/d, and high caffeine intake is above 500 mg/day [34]. And the definitions of drinking frequency are regular intake (every day and above 5 times per week) and rare intake (below 2 times per week and never drinking). In line with data from included research showing an inverted U-shaped caffeine dose-response curve [20, 22, 25, 27], regularly intaking moderate caffeine had a better cognitive function and a lower AD risk. However, low caffeine intake levels had a borderline positive or null relationship with AD risk [27, 28], and high caffeine consumption may increase the risk of AD and decrease cognitive performance, especially from coffee intake [27].

Additionally, as the long-term follow-up observations were commonly used in the cohort design. And coffee drinking habits may alter over time, possibly after a significant interval, if the cognitive state or other environmental influences such as lifestyle changes occur. The more obvious protective effects of caffeine from coffee against AD were more likely to be reported in studies with shorter follow-up as cognition-impaired patients would reduce their daily coffee intake compared to healthy participants, just like the study conducted by Lindsay et al. of 5 years [20] compared with that by Larsson and Wolk of 12.3 years [28].

Coffee, which is more frequently consumed in Western countries than tea as a more popular beverage in Eastern countries, contains much higher amounts of caffeine than any type of tea [25]. Studies in Western countries consistently reported that coffee had neuroprotective effects but null associations of tea consumption, while Eastern countries found the opposite. The neuroprotective effects of tea consumption may be more related to the abundant tea flavonoids (catechins), especially EGCG in green tea and theaflavins in black tea, rather than the stimulant effect of scarce caffeine contents [32]. Furthermore, diversities connected to the ratio of tea leaves to hot water (the boiling way) and the reuse habits of the same tea leaves several times in East Asia in comparison with single-use coffee also make a difference in the analysis between the exact dosage of caffeine and positive neuroprotective effects [35]. And the amounts of caffeine intake from tea also depend on social and cultural diversities. For example, Japanese subjects consume vastly green tea (2 cups of green tea per day; one cup is 100 ml) as a social activity, while Chinese subjects consume a range of tea (more black/oolong tea) [4], resulting in inconsistent results about protective effects of different types of tea among Japanese and Chinese subjects. Furthermore, because Westerners consume more black tea and rarer green tea than Easterners, a European study observed that habitual black tea consumption could lower the risk of AD but the plateau effects were up to 200 ml/d, even corresponding to the neuroprotective effects of lower caffeine amounts but in a habitual intake. And, by meta-analysis, which predominately had summarized Chinese studies, partially due to neuroprotective effects of caffeine in tea, tea consumption could reduce the risk of cognitive disorders in elders compared to nonconsumers or rare consumers [33]. It is thus reasonably assumed that the true association and interplay of flavonoids and caffeine with neuroprotection effects were underestimated. And all the studies on the neuroprotective effects of tea but not coffee were cross-sectional studies, with the results confined by the inference of a temporal causal relation between coffee and tea consumption and prevalence of AD, but the cognition in the old adults is shaped by long-term exposures [4]. Furthermore, the exact neuroprotection of tea for the old adults has also been affected by ambiguous drinking history and durations of tea before the involvement of studies. Further studies are needed to gather data from the long-term consumption of caffeinated coffee and tea to confirm the dose- and frequency-dependent association and the exact time of caffeine when its neuroprotective benefits begin [24]. And more well-defined studies are thus needed to be conducted on different racial/ethnic groups to achieve greater biological plausibility.

Findings of human epidemiological studies may also be influenced by various residual confounders, including smoking and physical activities, as a result of measurement errors or complementary effects of other active substances in coffee or/and tea, including magnesium [36], EGCG [37], and theaflavins [38], indicating human studies did not support the role of caffeine isolation in AD prophylactics.

Additionally, observational studies focusing on data from the self-reported questionnaires easily introduced biases and created incorrect information, especially in the long-term study. For example, the variability of daily doses of caffeine is increased through occasionally intaking other sources of caffeine like cola without informing the researchers. These exposure misclassifications undermine the methodological approach in particular in recalling it in the long term [24].

Further, different meta-analyses applied different searching strategies, inclusion criteria, and methods to select data for quantitative analysis, resulting in inconsistent findings [24]. And the quality of primary studies is the validity of meta-analysis. None of the double-blind placebo-controlled trials was selected in the meta-analysis that could provide more robust evidence [29], while observationally epidemiological studies were included, which recruited various participants, used different sample sizes, and applied different diagnostic criteria and methods of data analysis. For example, CSHA was a nationwide population-based study [20] while the study of Maia and De Mendonça [22] was a small hospital-based study with only 108 participants. Kuriyama et al. [31] utilized a cutoff value of MMSE of 26 for cognitive impairment diagnosis compared with 23 in Ng et al. [4]. And meta-analysis has evened up a cup of coffee or tea among all studies, even though there were discrepancies in the definition of caffeine volumes in a cup, making it difficult to validate the dosages of neuroprotective effects of caffeine.

Besides, the main methodological limitation of this study was to neglect the effects of the coffee and tea preparation method, as well as specific coffee and tea types such as decaffeinated coffee and tea. Instead, it used 90 mg caffeine per 230 ml coffee, 15 mg caffeine per 100 ml green tea, and 17 mg caffeine per 100 ml black/oolong tea directly.

In conclusion, it is reasonably suggested that caffeine from moderate and regular caffeine consumption from coffee could impede AD progression but may not for tea intake. And it is required to conduct further well-defined studies on the exact optimal dosages, frequencies, and durations of caffeine from various tea types to minimize the risk of AD.

## 3. Caffeine: Pharmacokinetic Profile

The pharmacokinetic profile of caffeine may be linked to the favorable effects of caffeine on reducing the risk of AD.

After consuming caffeine, caffeine can be absorbed quickly and completely by the gastrointestinal tract, especially in the small intestine, with very high bioavailability (99%-100%) [39]. 96.34 mg of caffeine resulted in a maximal plasma concentration of 2.47 *μ*g/ml [40], in the following 30 to 60 minutes [41]. Due to the hydrophobic properties of caffeine, it can also cross through BBB quickly, and then, the brain achieves similar caffeine concentrations as blood, proposing mechanisms of neuroprotection against cognitive dysfunction by oral caffeine intake [41]. Long-term caffeine consumption leads to adaptive changes in the brain, indicating greater betterment on cognitive performance that occurred among the older adults with continuous and regular caffeine consumption [42]. And chronic caffeine treatments could protect against seizures and maintain spatial memory in the mouse model, which was greater than acute caffeine administration [42].

The elimination half-life of moderate amounts of caffeine in systemic circulation has been reported about 5 hours, indicating a quick metabolic rate of caffeine [43]. But the high doses of caffeine over 500 mg have lower elimination rates and thus may affect the cardiovascular system with their positive inotropic and chronotropic effects and the central nervous system with their locomotor activity stimulation and anxiogenic-like effects, accountable for tremor, tachycardia, and anxiety, respectively [34]. But in the habitual caffeine consumers with moderate amounts of caffeine consumption, the acute proarrhythmic effect even caused by high caffeine intake was somewhat attenuated [44]. But regularly intaking high amounts of caffeine leads to caffeine abuse and dependence and can result in caffeine intoxication, which puts individuals at risk for premature and unnatural death [34]. Consequently, caffeine is a central nervous stimulant and should not be used in excess. When used to treat AD, it may require controlling the doses of caffeine below 500 mg/d.

## 4. Pathogenesis of Alzheimer's Disease and Mechanisms of Caffeine Therapies

AD progressively causes neuronal damage and leads to dementia, which is commonly related to cognitive dysfunction and mental decline, being the third biggest cause of old disabilities and death [45]. This age-related problem is further influenced by population aging and leads to substantial growth in the AD patient population from 32.5 million in 2021 to 53.3 million by 2030 [46].

The neuropathological hallmarks of AD are the cerebral extracellular deposition of diffuse and neuritic senile plaques made by A*β* peptides, the intracellular aggregation of flame-shaped NFTs composed of hyperphosphorylated aggregates of the microtubule-associated tau protein, and the selectively large-scale neuronal loss [47]. In understanding the pathology, neurobiological mechanisms underlying AD have been the key. And the most important changes identified can be explained currently by A*β* theory, tau protein theory, oxidative stress theory, ApoE4 theory, and adenosine theory.

Meanwhile, human studies do not allow concluding on the role of caffeine itself in the modulation of AD risk. This article has concluded some experimental studies, especially in the transgenic mouse models of AD, based on the biological alternations observed in these human pathologies, to further investigate the effects of caffeine on AD development and potential therapeutic effects and dosages.

### 4.1. A*β* Theory

A*β* theory is related to the imbalance between the production of A*β* through proteolysis of APP by *β*-secretase and *γ*-secretase and the clearance of produced A*β*, which is the triggering event and the most important factor [47].

Newly produced A*β* comes into a dynamic equilibrium between isoforms soluble A*β*_1-40_ and deposited A*β*_1-42_ [48]. And the soluble A*β*_1-40_ can be cleared out of the brain and entered into plasma down a concentration gradient [48], while the deposited toxic A*β*_1-42_ is more difficult to be cleared due to greater hydrophobicity, which leads to acquiring the configuration of a *β*-pleated sheet and easily clumping themselves together to cause depositions of amyloid neuritic plaques, which disrupt cell functions and lead to AD [49]. By targeting *β*-secretase and *γ*-secretase to reduce A*β* production or increasing the clearance speed of deposited A*β*_1-42_, the progression of AD might be relieved.

**Figure 1:**
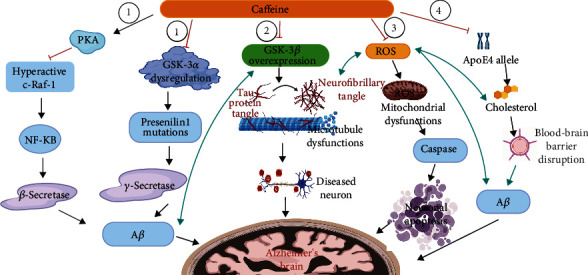
Caffeine neuroprotective mechanisms. (1) A*β* theory contains two routes. Firstly, caffeine stimulates PKA activity that decreases the hyperactive form of c-Raf-1. This abnormal c-Raf-1 form supports AD progression by activating the NF-*κ*B pathway and *β*-secretase expression. Secondly, caffeine lowers the GSK-3*α* dysregulation which increases PS1 mutation and *γ*-secretase expression. (2) Tau protein theory relates to the caffeine deactivating GSK-3*β* expression which can also be triggered by A*β* that expedites tau hyperphosphorylation and neurofibrillary tangle formation inside neurons. (3) Oxidative stress theory shows that caffeine inhibits ROS formation which can be promoted by A*β*. ROS can impair the mitochondrial electron transport system, further triggering caspase and neuronal apoptosis. (4) ApoE4 theory shows that caffeine can decrease high plasma and astrocyte cholesterol levels induced by high ApoE4 levels and reduce BBB disruptions by hypercholesterolemia (^1^*Adapted from* “*Pathology of Alzheimer*'*s Disease*”, *by BioRender*.*com* (*2021*). *Retrieved from*https://app.biorender.com/biorender-templates/t-5d8baeb4f7e1a5007dd46b18-pathology-of-alzheimers-disease/).

APPsw mice, which were the most prominent transgenic AD models in animals, can develop substantial levels of brain A*β* and widespread cognitive impairment with age [50]. The 4-5 weeks of treatment of 1.5 mg/d caffeine with the human equivalent of 500 mg/d caffeine in aged APPsw mice (18-19 months old) could stimulate PKA activity which would decrease the hyperactive form of c-Raf-1. This would correct dysregulation of the c-Raf-1 inflammatory pathway, inactivating the NF-*κ*B pathway and suppressing *β*-secretase expression (Figure 1, Pathway 1). Therefore, the evident A*β* deposition was reduced by 46% and 40% within the entorhinal cortex and hippocampus of Tg caffeine-treated mice compared to Tg controls in total, respectively, at 20–21 months of age [51]. Among them, soluble A*β*_1-40_ and insoluble A*β*_1-42_ levels of aged caffeine-treated Tg mice were reduced by 25% and 51% in the cortex and by 37% and 59% in the hippocampus, respectively, when compared with Tg controls [51]. Also, 1.5 mg/d caffeine treatment to aged 4-month-old APPsw Tg mice for 5.5 months could reduce *β*-secretase by 50% when following completion of behavioral testing at 9.5 months, then significantly lowering soluble A*β*_1-40_ production by 37% (*P* < 0.05) and insoluble A*β*_1-42_ production by 32% (*P* < 0.05) as compared to Tg control mice [41]. GSK-3*α* dysregulation is known to A*β* production by enhancing PS1 mutation which increases the *γ*-secretase cleavage of APP activity [52]. Caffeine (1.5 mg/d)-treated Tg mice had normalized PS1 band density ratios, compared with the significantly elevated Tg control group, after 5.5 months [41]. When treating cultured SweAPP N2a cells with caffeine in a dose-response manner (0-20 *μ*M), the maximal effects of decreasing active GSK-3*α* levels were achieved at 20 *μ*M (the human equivalent of 100–200 mg of caffeine) by 90 minutes [51] (Figure 1, Pathway 1).

### 4.2. Tau Protein Theory

Although A*β* theory is regarded to be the beginning of AD progression, however, it cannot fully explain the etiopathogenesis of AD. Tau protein is the secondary pathogenic event, subsequently leading to neurodegeneration [53]. A*β* exposure promotes GSK-3*β* overexpression, connected to neurodegeneration-related tau hyperphosphorylation [54]. Indeed, a study reported that chronic lithium (GSK-3*β* inhibitor) treatment prevented tau hyperphosphorylation in the GSK-3*β* transgenic mice [55].

Tau is a highly soluble protein whose biological activities are related to microtubules and are regulated by the degree of phosphorylation [56]. Under normal phosphorylation conditions, tau supports stabilizing the functions of microtubules on neuronal growth and axonal nutrient transport, while hyperphosphorylated tau loses its interactions with microtubules and prefers to aggregate with other tau molecules, forming neurofibrillary tangles inside neurons [56]. These neurofibrillary tangles consequently lead to microtubule dysfunction and blockage of the neuronal transport system, which damages the synaptic communications between neurons and AD-related brain changes [56]. And neurofibrillary tangles firstly found in the EC and hippocampus can extend to the amygdala and cortical areas (temporal, frontal, and parietal), causing more damage [57, 58].

The changes in A*β* oligomers and tau protein are reported by studies to be the most important factors for neuronal dysfunction in AD pathology [59, 60]. And the strategies refer to decreasing phosphorylation degrees of tau.

In SweAPP N2a cells, the best caffeine treatment for suppression of GSK-3*β* levels was 20 *μ*M for 30 minutes, and a lower phosphorylation degree of tau was proposed [51] (Figure 1, Pathway 2). 0.3 g/l of chronic caffeine delivery through drinking water (4 *μ*M plasma caffeine) to THY-Tau22 mice (aged 2 months old) for 10 months was significantly associated with an increase in dephosphorylated tau protein at Tau1 pathologic epitopes by 36.4% (±7.4%), as well as mitigated levels of proteolytic fragments of tau protein by reducing N-terminal fragments by 40.9% (±5.2%) and C-terminal fragments by 54.8% (±3.5%), as compared to untreated THY-Tau22 mice [61]. Reduction of tau phosphorylation by caffeine is consistent with an *in vitro* model of cultured cortical neurons (SH-SY5Y cells) in the nonpathogenic context, with dosages of 20 mM [62], which has been far higher than those achieved following habitual caffeine consumption (>10 mM) [63].

### 4.3. Oxidative Stress Theory

It is well understood that AD is strongly linked to extensive cellular OS [64]. OS is related to ROS accumulation in the brain because of inequality between ROS generation and antioxidant clearance activity [65]. The ROS could react quickly to biological components like lipid, leading to malfunction of the brain because the brain is mostly made up of a lipid that is easy to oxidize [65]. In addition, ROS could impair the mitochondrial electron transport system by disrupting its antioxidant enzyme functions, SOD1 and SOD2, causing a further increase in ROS levels that finally activate caspase and subsequently neuronal apoptosis [66] (Figure 1, Pathway 3). Also, OS could augment A*β* production and aggregation and facilitate tau hyperphosphorylation, which, in turn, further promotes ROS formation [67]. Thus, treatment with antioxidant properties could protect neurons from oxidative stress and A*β* toxicity.

Caffeine can be the antioxidant to inhibit lipid peroxidation and mitigate OS by suppressing the production of ROS [65]. The use of 10 *μ*M caffeine treatment might reduce intracellular ROS by 40.36%, increase SOD activity by 48.55%, and decrease malondialdehyde by 44.29% of the SH-SY5Y cells which have been exposed to the combination of A*β*_25-35_ and AlCl_3_ for 48 h, and antiapoptotic Bcl-2 protein levels for the prevention of neuronal death has been rescued [68]. Furthermore, the number of caspase-3-positive neurons was reduced by 48% after 1.84 mg/d caffeine treatment (equivalent to daily human consumption of 4.86 mg/kg body weight of caffeine) as compared to cultures treated with only 20 *μ*M of A*β*_25-35_ for 48 h, concurring the neuroprotective effects of caffeine against A*β*_25-35_-induced neuronal death [69].

### 4.4. ApoE4 Theory

ApoE4 is considered the largest genetic risk factor for AD, with a prevalence of about 14%, conferring a drastically elevated risk of AD with an earlier age of onset in a gene dose-dependent manner [70]. ApoE4 promotes the accumulation, aggregation, and deposition of A*β* in the brain. ApoE4 might be less efficient for clearing A*β* in the BBB due to a lower affinity to A*β* than other ApoE isoforms (ApoE2, ApoE3) [70].

Besides, ApoE4 also generates aberrant brain cholesterol metabolism which can further increase A*β* generation and contribute to the AD risk [70]. ApoE is mainly produced by brain astrocytes, which account for up to 40% of all brain cells, and could carry the lipoprotein-bound cholesterol from circulating plasma to the brain, which has been regulated by the presence of BBB [70]. ApoE4 is less efficient in transporting cholesterol from astrocytes to neurons and has a low binding capacity to plasma cholesterol [70]. Thus, high ApoE4 levels may lead to elevated cholesterol levels in the plasma and astrocytes [71]. And 2% cholesterol-enriched diets could induce hypercholesterolemia in rabbits; 3 times higher levels of insoluble A*β*_1-40_ were achieved by increasing *γ*-secretase activity to cleave APP on the hippocampus [72]. Hypercholesterolemia has been associated with OS by increasing ROS levels [72], and it could also disrupt BBB, increasing brain cholesterol levels further [73] (Figure 1, Pathway 4). Thus, strategies refer to reducing brain cholesterol accumulation.

0.5 mg/d and 30 mg/d caffeine treatments for 12 weeks decreased cholesterol-induced A*β* accumulation and increased the phosphorylated tau and active form of enzyme GSK-3*β*, as well as ROS generation in the hippocampus of rabbits (1.5-2 years old) which were daily fed a 2% cholesterol-enriched diet for 12 weeks. But the low caffeine dose (0.5 mg/d) was more efficient than the high dose (30 mg/d) in reducing A*β*_40_ and A*β*_42_ levels (-33.64% compared with -22.62%; -58.65% compared with -45.46%, respectively), which were reduced to similar levels as the control [72]. 12 weeks of 3 mg/d caffeine was given to rabbits aged 1.5 to 2 years, blocking the increased disruptions of BBB induced by the daily 2% cholesterol-enriched diet [73]. This was characterized by stabilization of the tight junctions between adjacent endothelial cells which involved an increase in expression of tight junction proteins including occludin and zonula occludens by 72.71% and 50.37%, respectively [73].

### 4.5. Adenosine Theory

Aside from the common molecular pathogenesis of AD and associated theories, where many distinct factors interrelate, caffeine is largely linked to adenosine theory, which also interacts with other theories.

Adenosine is an endogenous neuroprotectant abundant in the CNS, and its extracellular concentrations rise considerably in response to brain damage, neuroinflammation, and aging [74]. Adenosine effects are mediated by interactions with G protein-coupled receptors called adenosine receptors, such as inhibitory A_1_R and excitatory A_2A_R [75].

A_1_R is found in abundance in the neocortex, cerebellum, hippocampus, and dorsal horn of the spinal cord [76]. A_2A_R is extensively expressed in the striatopallidal neurons and olfactory bulb, with lesser levels in other brain regions like the hippocampus [76]. Because low concentrations of adenosine prefer to act on the A_1_R, while greater levels prefer to act on the A_2A_R, aging causes an imbalance in the expression of A_1_R and A_2A_R, contributing to cognitive impairment and an increased risk of AD [77, 78]. Meanwhile, adenosine lacks its inhibitory  A_1_R-mediated neuroprotective effects and ATP/adenosine metabolism in the aged brain but is modified to favor neurotransmission concerning stimulatory  A_2A_R; a physiological cost may be suggested by an increased vulnerability of senescent neurons to excitatory amino acid toxicity and a decrease in the number of functioning synapses [79]. Of high interest, A_2A_R antagonists, in particular, have been proposed to protect against cognitive and memory dysfunction evoked in experimental models of AD [41], independent of  A_1_R-mediated responses [80].

**Figure 2:**
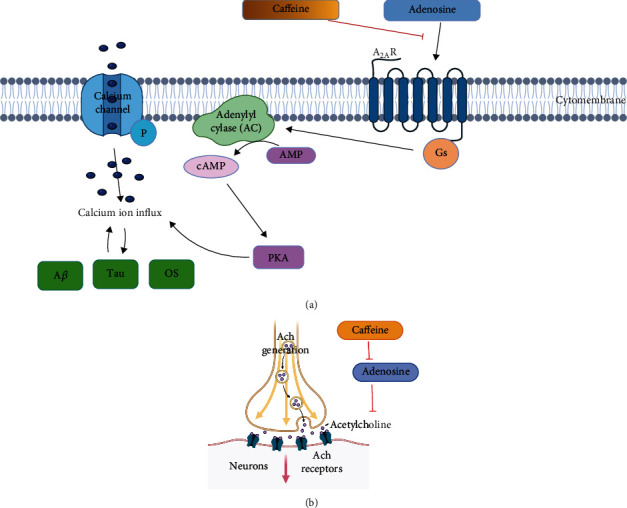
Caffeine intervenes with adenosine theory related to (a) lowering A*β* production by antagonizing A_2A_R which can increase AC levels, cAMP and PKA activities, and overload of intracellular Ca^2+^ (^2^*BioRender*.*com* (*2021*). [*Online*]. *Available from*: https://app.biorender.com/user/signin/) and (b) inhibit adenosine functions on the decrease of neurotransmitter Ach expression (created with https://biorender.com/ Created with BioRender.com.^3^*Adapted from* “*Neuromuscular Junction*”, *by BioRender*.*com* (*2021*). *Retrieved from*https://app.biorender.com/biorender-templates/t-5ed6b2d243ee8200b0135913-neuromuscular-junction/).

Furthermore, the activations and increased numbers of A_2A_R increase their coupling to G protein and efficacy in increasing AC levels, leading to AMP being converted to cAMP and higher levels of PKA. The calcium channels are more phosphorylated, resulting in an overload of intracellular Ca^2+^ [74], which stimulates A*β* and tau protein production and increases OS and neuroinflammation, ultimately contributing to increasing AD risk (Figure 2(a)) [64]. Also, the A*β* may promote overload of cellular calcium by inducing membrane-related OS and forming pores in the membrane [81]. Furthermore, the cholinergic and adenosinergic systems in the aged brain have an inverse relationship, with key neurotransmitter Ach levels in the brain declining with age while adenosine levels rise [82]. And because adenosine inhibits the release of Ach [83], adenosine accumulation has been linked to the progression of age-related cognitive deficits, making it an attractive target for pharmaceutical intervention (Figure 2(b)).

**Figure 3:**
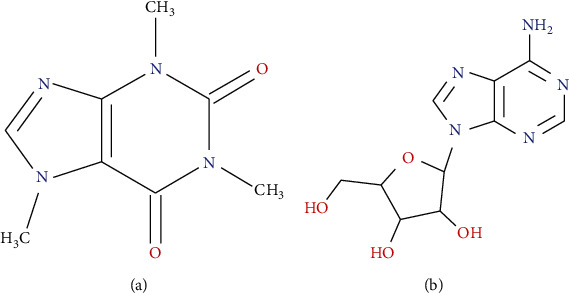
The chemical structure of caffeine (a) and adenosine (b) (^4^*KingDraw* (*2021*). [*Online*]. *Available from*http://www.kingdraw.cn/en/index.html/).

Caffeine, a well-known neuromodulator with an associative effect on cognitive performance, is structurally similar to adenosine due to purine backbones (Figure 3), which compete with the actions of adenosine as a nonselective A_2A_R antagonist [84].

Subchronic administration of daily 30 mg/kg caffeine for 4 days to mice (3-4 months old, 35-45 g) (the equivalent of 360-540 mg of caffeine) prevented A*β*_25-35_-induced amnesic effects [84], extending the finding that 25 *μ*M caffeine fully prevented the death of cultured cerebellar granule neurons of rats caused by the A*β*_25-35_ through stimulating the cholinergic neurotransmission [85]. Chronic administration of high amounts of caffeine (100 mg/kg/d) to mice (25-30 g) for 4 days resulted in a 40-50% increase in the density of cholinergic, muscarinic, and nicotinic receptors in the brain and may also have augmented cholinergic activity, which facilitated disruptions in the progression of AD [86].

Based on data collected from animal models and cell lines, chronic caffeine administration or other pharmacological agents that mimic caffeine in moderate amounts (200-500 mg/d) at midlife would have therapeutic potential in the AD treatment later in life according to five theories, especially attenuating the A*β* burden and A*β*-induced neurotoxicity. Even experimental studies indicate rather favorable effects of caffeine; such benefits may not be fully relevant to AD in humans, particularly when high dosages were used, necessitating us to carefully analyze and conduct more well-defined human studies to evaluate the role of caffeine on AD treatment. Meanwhile, a meta-analysis of diverse animal models also found that the effects of caffeine and A_2A_R antagonists are mostly determined by the dose, the schedule and time of administration, and the method of administration [87]. Moderate dosages of caffeine have been shown to increase memory function in mice [88–90], whereas greater doses of caffeine have been shown to damage memory acquisition [91, 92].

## 5. Conclusions

In conclusion, based on the results of epidemiological and experimental studies, moderate and regular caffeine consumption may help to prevent or delay the onset of AD and may be a viable therapeutic approach. However, before conducting rigorous preclinical and clinical research on its therapeutic potential in terms of precise neuroprotective dosages, frequencies, and durations, this recommendation would be premature. And to answer conflicting results in some human studies, the future study is required to set international consensual criteria for outcome measure, apply multivariate analyses to manage various confounding risk factors, clarify the drinking history of coffee and tea in the self-reported questionnaires, recruit a large number of participants from multiethnic backgrounds, and conduct a long follow-up period.

Meanwhile, as long as caffeine intake is maintained daily (e.g., tolerance), moderate usage of caffeine is usually not associated with harmful side effects. Although caffeine has been suspected of causing hypertension, there is no association between caffeine consumption in coffee or/and tea and blood pressure. Given the already widespread use and acceptance of coffee in moderate amounts, long-term coffee intake could be a viable strategy for reducing the risk of AD. However, more research into the effects of tea consumption on the risk of AD is needed.

## Figures and Tables

**Table 1:** The characteristics of selected human clinical trials (*N* = 15).

Author (year)	Country	Study design	Follow-up period (years)	Population	Caffeine intake, source	Outcome measure	OR, HR, or RR (95% CI)	Covariates
*Caffeine*								
Maia and De Mendonça (2002) [22]	Portugal	Case-control study	N/A	54 cases and 54 controls (matched for age and sex) Caucasians aged above 50 years	Daily caffeine consumption during the last 20 years preceded the diagnosis of AD 198.7 ± 135.7 mg per day consumed by healthy cases as compared to 73.9 ± 97.9 mg per day consumed by AD patients	AD (NINCDS-ADRDA)	Caffeine: 0.40 (0.25-0.67)	Age, sex, smoking habits (nicotine consumption), alcohol consumption, nonsteroidal anti-inflammatory drugs, heart diseases, family history of dementia, education, head trauma, stroke, diabetes, vitamin E, hypertension, gastric disorder
Gelber et al. (2011) [23]	USA	Case-control study Data derived from HAAS Programme	N/A	118 AD cases among 3494 Japanese American participants aged from 71 to 93 years	Caffeine: >115.5-188.0 mg/d vs. 0-115.5 mg/d >188.0-277.5 mg/d vs. 0-115.5 mg/d >277.5-415.0 vs. 0-115.5 mg/d >415.0-2673.0 mg/d vs. 0-115.5 mg/d	AD (NINCDS-ADRDA) Cognitive decline: CASI < 74	Caffeine: 1.20 (0.65-2.23) 1.15 (0.62-2.11) 1.07 (0.57-2.00) 0.95 (0.46-1.95)	Age, BMI, physical activity index, cigarette smoking, alcohol consumption, education, hypertension, education, elevated cholesterol, ApoE *ε*4 status, number of childhood years spent in Japan, history of diabetes mellitus, CVD, occupational complexity, and marital status
Santos et al. (2010) [24]	Europe, Australia, North America, Asia	Meta-analyses 11 selected studies (2 case-control, 9 cohort studies)	N/A	19,928 participants aged above 50 years	Caffeine intake	AD (NINCDS-ADRDA) and cognitive impairment (MMSE or Benton Visual Retention Test scores)	Cognitive impairment: 0.84 (0.72-0.99)	N/A
*Coffee*								
Eskelinen et al. (2009) [25]	Finland	Cohort study Data from the CAIDE study is within the North Karelia Project and the FINMONICA study	21	48 cases among 1409 Finns aged from 65 to 79 years	Coffee: 270-450 mg/d vs. 0-180 mg/d caffeine (3-5 cups/d vs. 0-2 cups/d) >450 mg/d vs. 0-180 mg/d caffeine (>5 cups/d vs. 0-2 cups/d) Tea: 17 mg/d caffeine (≥1 cup/d vs. none)	AD (NINCDS-ADRDA and MMSE ≤ 24)	Coffee: 0.42 (0.12-1.46) 1.01 (0.33-3.08) Tea: 0.91 (0.48-1.71)	Age, sex, education, the community of residence, follow-up time, midlife smoking, BMI, SBP, total serum cholesterol, ApoE *ε*4, physical activity, presence of late-life MI/strokes/DM, and Beck Depression Scale
Lindsay et al. (2002) [20]	Canada	Cohort study Data from CSHA	5	194 AD cases among 4615 Canadians aged above 65 years	Daily coffee (243 mg/d caffeine) [26] and tea (64 mg/d caffeine) consumption [19] as compared to no drinking	AD (NINCDS-ADRDA criteria and 3MS score < 78/100), *Diagnostic and Statistical Manual of Mental Disorders*, Fourth Edition, criteria	Coffee: 0.69 (0.50-0.96) Tea: 1.12 (0.78-1.61)	Age, sex, and education
Park et al. (2017) [27]	USA	Cohort study Data from the MEC study	16.2	1404 AD deaths among 185,855 Americans aged from 45 to 75 years Americans (African Americans, Native Hawaiians, Japanese Americans, Latinos, and whites)	Coffee: 90 mg/d vs. 0 mg/d caffeine (1 cup/d vs. none) 180-270 mg/d vs. 0 mg/d caffeine (2-3 cups/d vs. none) ≥360 mg/d vs. 0 mg/d caffeine (≥4 cups/d vs. none)	Death ascertainment by annual linkage to files of state death certificates in California and Hawaii and periodic linkage to the National Death Index AD death is defined as follows: ICD-9: 331.0 ICD-10: G30	Coffee: 0.90 (0.71-1.14) 1.16 (0.90-1.49) 1.33 (0.86-2.04)	Age, sex, race/ethnicity, education, cigarette smoking, preexisting chronic diseases, BMI, physical activity, alcohol consumption, total energy intake, and energy from fat
Larsson and Wolk (2018) [28]	Sweden	Cohort study Data from the National Research Infrastructure SIMPLER	12.6 years	1299 AD cases among 28,775 Swedish participants aged from 65-83 years	Coffee: 59-171 mg/d vs. <59 mg/d caffeine (1-2.9 cups/d vs. <1 cup/d) 177-289 mg/d vs. <59 mg/d caffeine (3-4.9 cups/d vs. <1 cup/d) ≥295 mg/d vs. <59 mg/d caffeine (≥5 cups/d vs. <1 cup/d) (1 cup = 150 ml)	N/A	Coffee: 0.90 (0.70-1.17) 1.01 (0.78-1.30) 0.93 (0.70-1.24)	Age, sex, education, smoking, BMI, exercise, walking or bicycling, history of hypertension, hypercholesterolemia, diabetes, sleep duration, alcohol
Quintana et al. (2007) [29]	Europe, North America, Australia	Meta-analyses 4 selected studies (2 case-control and 2 cohort studies)	N/A	5951 participants aged above 50 years	Coffee consumers vs. nonconsumers	AD (NINCDS-ADRDA)	AD: 0.73 (0.58-0.92)	N/A
*Tea*								
Chen et al. (2012) [30]	China	Case-control study Data from the third wave (2002) and fourth wave (2005) of CLHLS	N/A	1489 cases and 4822 Chinese controls aged above 65 years	Tea drinking habits	Cognitive decline: MMSE‐r ≤ 18	Tea: 0.82 (0.69-1.00)	Age, gender, marital status, financial status, residential area, BMI, hypertension, diabetes, smoking, alcohol, exercise habits, eating legumes and vegetables, fish, egg, meat, and sugar consumption
Kuriyama et al. (2006) [31]	Japan	Cross-sectional study	N/A	1003 Japanese participants aged above 70 years	Green tea: ≤45 mg/wk (≤3 cups/wk) 60-90 mg/wk or 15 mg/d (4-6 cups/wk or 1 cup/d) ≥30 mg/d (≥2 cups/d) Black or oolong tea: ≤51 mg/wk (≤3 cups/wk) 68-102 mg/wk or 17 mg/d (4-6 cups/wk or 1 cup/d) ≥34 mg/d (≥2 cups/d) Coffee: ≤117 mg/wk (≤3 cups/wk) 156-234 mg/wk or 39 mg/d (4-6 cups/wk or 1 cup/d) ≥78 mg/d (≥2 cups/d) (1 cup = 100 ml)	Cognitive impairment: MMSE < 26	Green tea: 1.0 (reference) 0.62 (0.33-1.19) 0.46 (0.30-0.72) Black or oolong tea: 1.0 (reference) 0.60 (0.35-1.02) 0.87 (0.55-1.38) Coffee: 1.0 (reference) 1.16 (0.78-1.73) 1.03 (0.59-1.80)	Age, sex, education, social activities, smoking, alcohol, physical activities, medical history, myocardial infarction, regular intake of supplements, self-rated health
Ng et al. (2008) [4]	Singapore	Cross-sectional study Data from SLAS	N/A	2194 Chinese living in Singapore aged above 55 years	Coffee: Never or rarely ≥84 mg/d caffeine (≥1 cups/d) Habitual tea intake (sum of English black tea, Chinese black or oolong tea, green tea): Low, medium, and high levels vs. no drinking Low: <37 mg/d caffeine (<1 cup/d) Medium: 37-185 mg/d caffeine (1-5 cups/d) High: ≥222 mg/d caffeine (≥6 cups/d) (1 cup = 215 ml)	Cognitive impairment: MMSE‐r ≤ 23	Coffee: 0.99 (0.69-1.45) Tea: Low: 0.56 (0.40-0.78) Medium: 0.45 (0.27-0.72) High: 0.37 (0.14-0.98)	Age, sex, education, cigarette smoking, alcohol consumption, vegetable and fruit consumption, fish consumption, BMI, hypertension, diabetes, heart disease, stroke, depression, ApoE *ε*4, physical activities, social and productive activities, tea consumption (for coffee), and coffee consumption (for tea)
Feng et al. (2010) [32]	Singapore	Cross-sectional study Data came from SLAS	N/A	716 Chinese participants aged from 55 to 88 years	Tea (include English black tea, Chinese black/oolong tea, and green tea): Never or rarely <37 mg/wk (<1 cup/wk) 37-222 mg/wk (1-6 cups/wk) 37-74 mg/d (1-2 cups/d) ≥111 mg/d (≥3 cups/d) Coffee: Never or rarely <84 mg/wk caffeine (<1 cup/wk) 84-504 mg/wk caffeine (1-6 cups/wk) 84-168 mg/d (1-2 cups/d) ≥252 mg/d (≥3 cups/d) (1 cup = 215 ml)	Neuropsychological and cognitive test battery: MMSE < 24	N/A	Age, sex, education, cigarette smoking, alcohol consumption, vegetable and fruit consumption, fish consumption, coffee consumption, medical conditions, blood pressure, fasting blood glucose, weight and height, ApoE *ε*4, physical activities, social activities, productive activities
Nurk et al. (2009) [21]	Norway	Cross-sectional study Part of population-based HUSK	N/A	2031 Norwegians aged from 70 to 74 years	Habitual tea intake during the previous year (mean value: 222 ml/d) vs. none	Cognitive impairment: m-MMSE score < 10	Tea: 0.95 (0.68–1.33)	Sex, history of CVD, diabetes, education, smoking status, use of vitamin supplements, and total energy intake
Ma et al. (2016) [33]	Asia, Europe, Australia, and North America	Meta-analyses 26 selected studies (10 case-control, 4 cohort, and 12 cross-sectional studies)	N/A	52,503 participants aged above 50 years	Daily tea consumption vs. nonconsumers/rare consumers	AD (NINCDS-ADRDA) and cognitive impairment (MMSE) Cognitive disorders include AD and cognitive impairment	Cognitive disorders: 0.65 (0.58-0.73) AD: 0.88 (0.65-1.12) Cognitive impairment: 0.52 (0.43-0.62)	N/A
Kim et al. (2015) [5]	Asia, Europe, Australia, North America	Meta-analyses 20 selected studies (5 case-control, 9 cohort, and 6 cross-sectional studies)	N/A	31,479 participants aged above 49 years	Caffeine intake	AD (NINCDS-ADRDA) and cognitive impairment (MMSE)	Cognitive disorders: 0.82 (0.67-1.01) AD: 0.78 (0.50-1.22) Cognitive impairment: 0.79 (0.61-1.04)	N/A

## Data Availability

After electronic searches on databases PubMed and ScienceDirect, potential eligible studies from 2000 up until 2020 have been identified. According to instructions in Boolean operators and wildcards, the searches applied the following terms to clarify dietary risk factors (coffee OR tea OR caffeine) combined with terms of interested results (cognit^∗^ AND (declin^∗^ OR damag^∗^)) or (neurodegenerat^∗^ OR Alzheimer^∗^). The range of obtained results is around 2000 records. After scanning titles, keywords, and the gist of abstracts in each article, the articles were retained for close reading and analysis of details if all of the following inclusion criteria are met: (1) the published paper had full length and was in a peer-reviewed source; (2) it evaluated caffeine which was sourced from caffeine, coffee, or tea; and (3) it mentioned AD, cognitive impairment, or cognitive decline. And a study was excluded if it met one or more of the following exclusion criteria: (1) the published paper was in a non-peer-reviewed source (i.e., website, magazines); (2) it was in the abstract form; (3) the investigational product was not caffeine, coffee, or tea; (4) it investigated diseases which were not related to cognitive disorders; and (5) it was a duplicate publication. In addition, the present article included several secondary research papers (i.e., narrative review, systemic review, and meta-analysis studies) which could recommend other relevant research studies with the same topics after looking through their reference lists as key clues. Articles in which caffeine was not studied were excluded. Articles, where sources of caffeine were not from coffee or tea, were excluded. Also, articles in which cognitive decline or Alzheimer's disease was not mentioned were excluded as well. Researchers paid deliberate attention to papers which concluded human studies or animal studies for neuroprotective effects of caffeine for approving arguments as well as theories behind the pathogenesis of neurodegenerative diseases. This paper focused on the prevention and postponement of progression of age-related neurodegenerative diseases; thus, analyses ignored cognitive decline within the normal range. And articles concerning the dosage and frequency of coffee and tea consumption were selected to have a deep analysis for comparing the difference between coffee and tea. This article includes both single studies like longitudinal studies and meta-analyses for more prudent considerations.

## Abbreviations

AD: Alzheimer's disease
CNS: Central nervous system
mg/d: Milligram per day
wk: Week
OR: Odds ratio
CI: Confidence interval
RR: Relative risk
*I*^2^: Heterogeneity
HR: Hazard ratio
MMSE: Mini-Mental State Examination (higher MMSE scores mean higher cognitive function), which measures global cognition including memory, attention, language, praxis, and visuospatial ability [24]
MMSE-r: The Chinese revised version of MMSE
m-MMSE: A modified version of the Mini-Mental State Examination
*B*: Regression coefficient
SE: Standard error
NINCDS-ADRDA: National Institute of Neurological and Communicative Disorders and Stroke and the Alzheimer's Disease and Related Disorders Association
BMI: Body mass index
HAAS Programme: Honolulu-Asia Aging prospective cohort Study Programme
CLHLS: Chinese Longitudinal Health Longevity Study
CAIDE: Cardiovascular Risk Factors, Aging and Dementia
SBP: Systolic blood pressure
MI: Myocardial infarction
DM: Diabetes mellitus
CSHA: Canadian Study of Health and Aging
MEC: Multiethnic cohort
SIMPLER: Swedish Infrastructure for Medical Population-based Life-course Environmental Research, previously the Swedish Mammography Cohort and the Cohort of Swedish Men
SLAS: Singapore Longitudinal Aging Study
HUSK: Hordaland Health Study
CASI: Cognitive Abilities Screening Instrument
ICD: Codes from the International Classification of Diseases
ICD-9: Ninth revision
ICD-10: Tenth revision
3MS: Modified Mini-Mental State Examination
d: Day
USA: The United States of America
N/A: None/anonymity
CVD: Cardiovascular disease
BBB: Blood-brain barrier
Ca^2+^: Calcium ions
EGCG: Epigallocatechin gallate
A*β* peptides: Amyloid beta peptides
NFTs: Neurofibrillary tangles
ApoE: Apolipoprotein E
APP: Amyloid precursor protein
*β*-Secretase: Beta-secretase
*γ*-Secretase: Gamma-secretase
APPsw: Swedish mutation
PKA: Protein kinase A
NF-*κ*B: Nuclear factor kappa-light-chain-enhancer of activated B cells
GSK-3: Glycogen synthase kinase-3
EC: Entorhinal cortex
OS: Oxidative stress
ROS: Reactive oxygen species
SOD1: Superoxide dismutase 1
SOD2: Superoxide dismutase 2
Ach: Acetylcholine
A_1_R: Adenosine A_1_ receptor
A_2A_R: Adenosine A_2A_ receptor
AC: Adenylyl cyclase
cAMP: Cyclic AMP
Tg: Transgenic
PS1: Presenilin 1
THY-Tau22: Characterized by a significant tau expression in the hippocampal formation with a small cortex pathology and no significant spinal cord pathology, making it a reliable model for assessing the modeling effects on hippocampal tau pathology and their associated effects on behavior and plasticity [61]
Malondialdehyde: A marker of oxidative stress.
